# Liberals lecture, conservatives communicate: Analyzing complexity and ideology in 381,609 political speeches

**DOI:** 10.1371/journal.pone.0208450

**Published:** 2019-02-06

**Authors:** Martijn Schoonvelde, Anna Brosius, Gijs Schumacher, Bert N. Bakker

**Affiliations:** 1 School of Politics and International Relations, University College Dublin, Dublin, Ireland; 2 Amsterdam School of Communication Research, University of Amsterdam, Amsterdam, Noord-Holland, the Netherlands; 3 Department of Political Science, University of Amsterdam, Amsterdam, Noord-Holland, the Netherlands; Saint Peter’s University, UNITED STATES

## Abstract

There is some evidence that liberal politicians use more complex language than conservative politicians. This evidence, however, is based on a specific set of speeches of US members of Congress and UK members of Parliament. This raises the question whether the relationship between ideology and linguistic complexity is a more general phenomenon or specific to this small group of politicians. To address this question, this paper analyzes 381,609 speeches given by politicians from five parliaments, by twelve European prime ministers, as well as speeches from party congresses over time and across countries. Our results replicate and generalize earlier findings: speakers from culturally liberal parties use more complex language than speakers from culturally conservative parties. Economic left-right differences, on the other hand, are not systematically linked to linguistic complexity.

## Introduction

Many have ridiculed Donald Trump for his use of simple language with low levels of linguistic complexity. For example, during the 2016 primaries, the Washington Post reported that Trump, based on a linguistic analysis of his speeches [1], “speaks like a 5th-grader”, while other politicians used language as complex as that of 6th—8th graders. Beyond its headline-grabbing appeal, this finding speaks to the more general claim that conservative politicians use simpler, less complex language than liberals. The unique rhetorics of Trump aside, this claim is backed up by evidence from US Senators and British members of Parliament [2–4]. Their divergence in linguistic complexity is argued to be rooted in personality differences among conservative and liberal politicians. The former prefer short, unambiguous statements, and the latter prefer longer compound sentences, expressing multiple points of view. In other words, liberals lecture (use complex language) and conservatives communicate (use simple language).

If such linguistic patterns are generalizable they should extend beyond specific American and British examples to other speakers, political systems and time periods. In this paper, we find results in support of such a general trend between political ideology and linguistic complexity. We analyzed 381,609 political speeches, including parliamentary speeches, party congress speeches and speeches from government leaders from Germany, Spain, the United Kingdom, Sweden and the Netherlands, spanning several decades. In multiple countries, we replicate the finding that speakers from culturally liberal parties use more complex language than speakers from culturally conservative parties. However, we find no systematic differences in language complexity between economically left- or right-wing, or opposition- and government politicians.

## Ideological differences in complexity

The way we speak reflects—to a degree—who we are [5]. Our linguistic habits, the words we use, and the grammatical choices we make are relatively stable over time and across contexts [6]. For example, Pennebaker and colleagues [7] analyze how often people use articles, prepositions and pronouns, as well as broader linguistic concepts such as emotional words, causation words, and words indicating social processes. They conclude that in texts as diverse as daily diaries from substance abuse patients, daily writing assignments from students, and journal abstracts from social psychologists, stable linguistic habits can be observed. Language complexity elicits such ‘psychometric properties’ as well (i.e., stability over time and across contexts). For example, research on linguistic habits of American and British politicians shows that conservative politicians make less complex statements than liberal politicians [3, 4, 8]. Cichocka *et al*. [9] show that the speeches of liberal US presidents score higher on integrative complexity than those of conservatives, as measured by the presence of “words involved in differentiation (exclusive words, tentative words, negations) as well as integration of different perspectives (conjunctions)” (p. 809). Conservative political bloggers use less complex language than their liberal counterparts [10] and conservative citizens use language that scores lower on integrative complexity than liberal citizens [11]. The only study outside of the Anglo-Saxon context finds that politicians from the Alternative for Germany—a populist, culturally conservative party—use simpler language than mainstream politicians [12].

But what is the reason for such linguistic differences among liberals and conservatives? Psychological research finds that liberals and conservatives vary in regard to their cognitive, affective, and motivational functioning [9]. This is expressed by differences in personality. For example, liberals are “generally more open-minded in their pursuit of creativity, novelty, and diversity, whereas conservatives’ lives are more orderly, conventional, and neat” [13]. In terms of the famous Big Five personality dimensions, liberals score higher on openness to experience and lower on conscientiousness than conservatives [14, 20]. These personality differences express themselves in various linguistic habits. For example, people high on openness to experience use more tentative words and longer words [7], people low on extraversion prefer rich vocabulary and use more formal language [15], people high on conscientiousness dislike using discrepancies (should, would), causation and exclusive words [16]. Conservatives also score higher than liberals on need for closure, which reflects preferences for reducing ambiguity and uncertainty [17]. By consequence, conservatives prefer using nouns over verbs and adjectives, because they convey more certainty [9]. They may also prefer shorter and clearer sentences. Compound sentences with multiple clauses, on the other hand, are more likely to convey ambiguity, and may thus appeal more to liberals who are generally more open-minded and tolerant of ambiguity.

Such patterns are plausible in the American context, but the extent to which they transcend is unclear. The work discussed so far relies heavily on a one-dimensional, conservative-liberal conceptualization of ideology [18]. The terms liberal and conservative, however, do not travel well across the Atlantic, and mean different things in Europe and the US. What is more, European politics is generally characterized by political competition along two dimensions, rather than just one [19]: a sociocultural conservative-liberal dimension and an economic left-right dimension. The former dimension typically includes issues like European integration, immigration and the environment [19]. The Dutch party system, for instance, includes cultural conservative parties with an economically moderate agenda (most prominently Geert Wilders’ Freedom Party), and cultural liberals with a right-wing (D66) or left-wing (Green Left) economic agenda. In our view, linguistic complexity is most likely related to the sociocultural liberal-conservative dimension because personality traits such as openness to experience, conscientiousness [14, 20], need for closure [21], authoritarianism and need for cognition [22] are more strongly associated with social conservatism than with economic conservatism. Across contexts, we expect the language of culturally conservative politicians to be less complex than the language of culturally liberal politicians. The associations between economic left-right ideology (or economic conservatism) and traits such as openness to experience, conscientiousness, need for structure and the value of conformity and security have been found to be much more dependent on voter and country characteristics [20, 21, 23]. As such we expect the economic left-right dimension to be less consistently associated with complexity.

### Other factors that explain linguistic complexity

Beyond ideology, contextual factors may also influence complexity of language. For example, speeches by American presidents have become simpler over time because they became more directed toward the public rather than a small political elite [24–27]. Increased media attention also demands less complex language. Rather than a linear time trend, the complexity of speech may vary depending on the economic and social context of the time. Philip Tetlock and colleagues [4] describe how differences in the complexity of speech between liberals and conservatives fluctuate. For example, Democrats deliver less complex speeches in a Republican-dominated Congress [4]. Other examples for this phenomenon include the decrease in the integrative complexity of statements by Tony Blair and George W. Bush after the 9/11 terrorist attacks [28] and New York mayor Rudolph Giuliani’s simpler language during times of crisis [29]. Furthermore, incumbency itself seems to increase speech complexity. US-American presidential candidates use more complex language once elected [2] and MPs of the governing party in the Canadian House of Commons systematically use more complex language than MPs of opposition parties [30]. In order to account for these factors, we add time and government-oppositions status of the party of the speaker as control variables to our models.

## Methods

Our analysis relies on three dataset: (1) ParlSpeech [31], (2) EUSpeech [32, 33] and (3) a dataset of party congress speeches [34]. Combined, these datasets contain speeches from 10 European countries and span a long period of time (up to a maximum of 70 years, between 1945-2015). The different corpora contain speeches targeted at various audiences: members of parliament (Parlspeech); partisans and party members (party congress speeches); ordinary voters and various political and societal elites (EUSpeech). This diverse corpus of speeches allows us to evaluate the generalizability of the claim that liberals use more complex language than conservatives. Tables A.1 through A.8 in S1 Appendix. contain (standardized) descriptive statistics for all corpora.

The ParlSpeech [31] dataset contains parliamentary speeches from seven European parliaments, fully covering periods of up to 28 years. It is a full sample of all available speeches in the different parliaments; thus, they cover a wide variety of topics and speakers. For the present study, we include speeches from the British House of Commons (*N* = 161,683, 1988–2015), the German Bundestag (*N* = 66,061, 1991–2013), the Dutch Tweede Kamer (*N* = 48,546, 1994–2015), the Spanish Congresso de los Disputados (*N* = 35,986, 1989–2015), and the Swedish Riksdag (*N* = 72,999, 1991–2015). All speeches were delivered in the country-specific language, and transcribed verbatim. In order to exclude interruptions, we only consider speeches with more than ten sentences of at least five words. We also exclude all chair(wo)men speeches, since they mostly serve to organize the debates (e.g. by announcing speakers), and are therefore structurally different from other speeches.

The EUSpeech dataset [32, 33] consists of all publicly available speeches from elites in the main European institutions, the IMF, and speeches of prime ministers—or president in the case of France—of 10 EU member states for the period ranging from early 2007 to late 2015. These countries are Czech Republic, France, Germany, Greece, Netherlands, Italy, Spain, United Kingdom, Poland and Portugal. For the analysis in this paper, we use all English-language prime minister (PM) speeches in this corpus. The speeches target various audiences: MPs, party members, interest groups, public officials, foreign officials, or citizens at rallies or events. The number of speeches we analyze per country varies between 63 in Italy and 787 the United Kingdom, amounting a total of 1847 (see S1 Appendix). Since we had only very few English speeches for Italian Prime Minister Prodi (3 speeches) and Portuguese Prime Minister Pedro Passos Coelho (6 speeches), we excluded them from this analysis.

The third dataset contains speeches at party congresses in Denmark and the Netherlands, covering the time period 1945–2017 [34]. We analyze 528 speeches from Denmark for the following parties: Danish People’s Party (*N* = 32), Unity List (11), Social Democrats (228), Socialist People’s Party (56), and Venstre (the Liberal Party, 201). We analyze 659 speeches from the Netherlands for the following parties: Socialist Party (16), Green Left (31), the Labour Party (187), VVD (the Liberal Party, 112), Christian Democratic Appeal (154), D66 (105) and the Freedom Party (8). We combined speeches from Christian Democratic Appeal congresses and those of the three constituent parties ARP, CHU and KVP. Furthermore, since the Freedom Party does not have a party organization in the traditional sense (in fact, it only has one member), our analysis included speeches delivered at meetings aimed to present the party and its (new) MPs, as these events are closest in form to a traditional party congress. In general, the majority of speeches are delivered by the party leader, the party chair, and other prominent party members. Nowadays, party congresses usually take place on an annual basis, with additional, extraordinary congresses during times of election. In the past, party congresses were more likely to take place on a bi-annual basis. The function of a party congress differs between parties and has changed over time [35]. For our purposes, the most important feature of these congresses is that the party leader or leaders give a speech to party members reporting on the party’s current and future activities. Such speeches typically contain sections on policies and policy-making, on party strategy and coalition possibilities, and also on the performance of the party itself. These speeches are delivered with different goals: to strengthen the internal cohesion of the party, to signal policy priorities to policy activists or alert voters, or to communicate strategic intentions to other parties. These speeches are public and it is likely that journalists report on them. This corpus is particularly interesting because of the various publics involved: party members, other parties, and voters. Speakers at party congresses have more agency regarding the topics of their speech than MPs, as they are not responding directly to someone, nor are they part of an ongoing debate.

### Method and variables

In order to analyze complexity over a large corpus of speeches across time and countries, automated methods are a necessity (NB: the data and scripts required to replicate the findings reported in this paper are posted on Harvard’s Dataverse: https://dataverse.harvard.edu/dataset.xhtml?persistentId=doi:10.7910/DVN/S4IZ8K). Most commonly, linguistic complexity is measured as an index of the average number of words per sentence and the average word length. The Flesch-Kincaid grade score is an example of such a measure of complexity [36]. It was initially developed by education researchers to score readability of a text, expressed as the years of schooling required to understand a given text without difficulty. It weighs average sentence length and average word length in a text as follows: $0.39\times \left(\frac{total\;words}{total\;sentences}\right)+11.8\times \frac{total\;syllables}{total\;words}-15.59$. Higher Flesch-Kincaid scores correspond to higher complexity, as a function longer words, longer sentences or both. In addition to education research, Flesch-Kincaid measures have been used in various others fields of study for a wide variety of research questions. In journalism research, a recent study shows that newspaper articles tend to be so complex that they are hardly understandable for a majority of readers [37]. Political scientists have found that people are less likely to vote on ballots that have more complex language [38]. Furthermore, political science textbooks have become more difficult to read over time [39], while political science journal articles tend to be relatively complex but not much more complex than a judicial opinion or an op-ed in the New York Times [40]. Moreover, survey questions that are formulated in a more complex manner, tend to result in more “don’t know” answers [41]. The Flesch-Kincaid readability score can be systematically applied to a large corpus of speeches. Furthermore, since it is a weighted average of word length and sentence length it also speaks to measures of cognitive and integrative complexity which are often used in psychology. The reason for this is that these measures increase with an increasing number of clauses in a compound sentence. Integrative complexity concerns the degree to which a text incorporates different viewpoints and integrates them. Traditionally it is scored using trained coders. Efforts to automate measurement of integrative complexity [42], have been met with considerable criticism [43], and we don’t know of validation efforts of measuring integrative complexity in different languages. A broader construct than integrative complexity is cognitive complexity or the degree of multidimensional, differentiated thinking revealed in a text. If a speaker or author gives several perspectives on a given topic, a text becomes cognitively more complex [7]. It is measured through a tally of exclusion words such as ‘but’, ‘without’ and ‘exclude’, as well as conjunctions such as ‘also’, ‘and’ and ‘although’. Similarly, words such as ‘may’, ‘possibly’, ‘sometimes’ have been argued to high cognitive complexity, and ‘always’, ‘only’ and ‘without a doubt’ low cognitive complexity. See for more discussion on various forms of complexity [43] Our approach does impede a comparison between countries, because languages may systematically differ in their complexity. It should be noted, however, that our comparisons are within countries, not across countries. Last, we note that there are other measures for linguistic complexity, tailored to specific languages such as the *Lesbarkeitsindex* (LIX) in German and the *Flesch-Douma* index in Dutch. However, we prefer using one measure for complexity across languages. Moreover, *Lesbarkeitsindex* and *Flesch Douma* correlate very strongly (*r* = 0.99) with Flesch Kincaid in the German and Dutch sections of our corpora.

Our unit of analysis is the individual speech. Our dependent variable is linguistic complexity measured by the Flesch-Kincaid Grade Level. The use of the Flesch-Kincaid scores—and other, similar measures—is very common in the study of political speeches. Flesch-Kincaid scores have been used to analyze famous political speeches—such as General McArthur’s farewell speech to the US Congress [44]—and to describe how politicians discuss policy reforms [45]. Others have used Flesch-Kincaid scores to test whether politicians competing in elections differ in the language they use. For example, Donald Trump uses much simpler language than Hillary Clinton [46–48]. But researchers found no meaningful differences in the speech complexity of Republican candidate Eisenhower and Democratic candidate Stevenson [49] and between Stevenson’s speeches in the 1952 and 1956 presidential races [50]. Sigelman [25] shows that U.S. inaugural speeches have become less complex with time. While George Washington’s inaugural address was very complex, George Bush’s 1989 inaugural address was far less complex. Elvin Lim arrives at a similar conclusion: he finds that presidential speeches were relatively complex in the eighteenth and nineteenth century but have become much simpler in recent decades [26]. This pattern of decreasing complexity is not limited to the United States but was found in speeches of Australian politicians as well [51]. Others have even used Flesch-Kincaid scores to show that when speeches of US presidents becomes simpler, this is associated with the use of more executive orders [52].

Contributing to the validity of the Flesch-Kincaid scores for measuring language complexity, Merry [53, p.64] found that the Flesch-Kincaid scores “correspond(s) to the complexity of the content of communications; statements with low grade levels are fairly basic, while those with high grade levels are more difficult to understand.” More recently, studies used Flesch-Kincaid scores to show that when politicians speak to their constituents, they tailor their speech to their constituents’ linguistic skills. In other words, politicians use simpler language when appealing to less educated constituents with fewer linguistic skills [27, 54]. Along these lines, Flesch-Kincaid scores have been used to make the point that during WWII, U.S. President Roosevelt and Australian President Curtis “developed political communication to create the resemblance of a closer relationship between the nation’s leader and citizens” [55, p. 77]. These studies illustrate that there is a long lasting and varied literature that uses Flesch-Kincaid scores to study political speeches.

The use of Flesch-Kincaid scores to measure the complexity of political text is not uncontested. Very recently, Benoit, Munger and Spirling [56] introduced a promising new domain-specific approach to measuring political sophistication in text. Their approach—which relies on crowd coders evaluating the difficulty of a large number of text snippets—accounts for statistical uncertainty and allows for comparability of various texts on a “political sophistication” scale. While we think this measure is very promising, it is not feasible for our project to determine textual complexity by crowdsourcing textual snippets to people in the Netherlands, Denmark, Sweden, Great Britain and Spain. Furthermore, we note that Benoit, Munger and Spirling find Flesch Reading Ease (FRE) to be a crucial predictor of sophistication: with that score alone they can correctly predict 72% of the human coders’ judgements of the most difficult text among two text snippets. The introduction of various additional text features (such as word rarity in the Google books corpus and the proportion nouns) only marginally improves on the prediction capacity of FRE alone.

**Fig 1:**
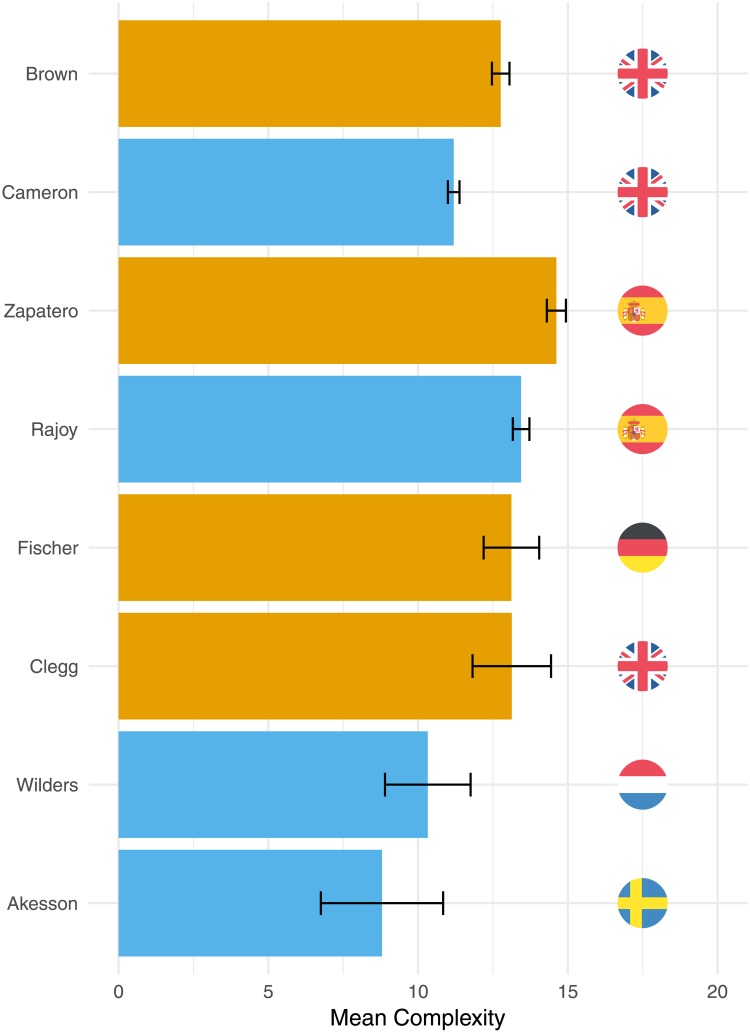
Descriptive information on linguistic complexity. The bars in this figure denote mean complexity scores, with 95% confidence intervals, of selected speakers.

Fig 1 presents mean, unstandardized complexity scores for a number of selected speakers. Gordon Brown (liberal), for example, gave speeches with much higher complexity scores than his successor David Cameron (conservative). For illustrative purposes, Table 1 contains two text snippets of Brown and Cameron talking about similar themes (Make Poverty History and the Help to Buy scheme) as well as their accompanying Flesch Kincaid grade levels. When reading the two snippets, it becomes clear that Brown’s speech is linguistically much more complex (Flesh-Kincaid of 19.5) than the speech of Cameron (Flesh-Kincaid score of 7). Most importantly, the Brown text consists of just one long sentence whereas the Cameron text contains multiple short sentences. Turning to the Spanish example in Fig 1, we see a similar pattern: the language of the liberal Prime Minister José Zapatero is more complex than that of his successor, the conservative Mariano Rajoy. To further illustrate our point, Fig 1 also projects the complexity of two liberal politicians, namely Joschka Fischer—key figure of the German Green Party and Minister of Foreign Affairs (1998-2005)—and Nick Clegg—the leader of Liberal Democrats (2007-2015) and deputy PM (2010-2015) in the UK—as well as two conservative politicians, namely Geert Wilders—the leader of the radical-right Freedom Party in the Netherlands (2005-now)—and Jimmie Åkesson—the leader of the radical-right Sweden Democrats (2005-now). The two liberal politicians (Fischer and Clegg) score notably higher on speech complexity than the two selected conservative politicians (Wilders and Åkesson). These examples also illustrate notable differences between countries. The Spanish Prime Ministers Rajoy and Zapatero score higher on complexity than the politicians from the UK, Netherlands and Sweden in this example: this could mean that they use more complex language but it could also signal that the two languages differ structurally in their complexity.

**Table 1:** Average speech complexity of selected speakers. This table contains example texts of David Cameron and Gordon Brown with accompanying Flesch-Kincaid (FK) grade level scores.

Speaker	Text	Flesch Kincaid score
G. Brown, 16 May ‘08	And I believe that no injustice will last forever, so people who are oppressed need not any longer journey without hope. And with this most powerful peaceful weapon for change: of conscience linked to conscience—people with a shared moral sense and a capacity to communicate and organize; and the power that comes from calling, networking, marching for change, millions can now be moved to action—as with Make Poverty History—against the great injustices of poverty, disease and environmental degradation.	19.5
D. Cameron, 13 Nov ‘13	Some people thought it might only benefit those buying relatively expensive properties. Again, that’s not the case. The typical property being bought under this scheme is around the average house price in the UK. So this is a successful scheme. Those are the numbers, but this is about more than numbers. It’s about hard-working people achieving their dream of home-ownership. And it’s part of the government’s long-term plan for getting our economy moving.	7.0

In our statistical models, we regress speech complexity on the following independent variables: left-right ideology, liberal-conservative ideology, a measure for time, and a dummy for speakers from the government party. The two ideology measures are taken from the Manifesto Project Database. This group systematically hand-coded quasi-sentences in the election manifestos of parties. Their codebook distinguishes in total 53 issues, of which most reflect a position on an issue. For example, quasi-sentences can be coded to reflect an anti-immigration or pro-immigration position. The salience of these opposite positions in the election manifesto can then be used to construct a scale that reflects a party’s position on immigration. Likewise, more inclusive scales can be constructed by combining several related issues. We followed this logic to construct a cultural liberal-conservative scale. Specifically, we sum attention to the conservative issues in the dataset (specifically, these are anti-EU, anti-immigration, pro-national way of life, pro-traditional morality, anti-multiculturalism, pro-military, anti-internationalism, pro-Freedom and Human Rights and pro-political authority, pro-law and order), log-transform them, and subtract the log-transformed sum of the attention to liberal issues in the dataset which mostly reflect opposites of the conservative issues. This entails pro-EU, pro-immigration, anti-national way of life, anti-traditional morality, pro-multiculturalism, anti-military, pro-internationalism, anti-imperialism, pro-peace, pro-environment, pro-culture, and support for under-privileged minority groups. A similar procedure was followed to create an economic left-right position. Left-wing items are market regulation, economic planning, corporatism, protectionism: positive, Keynesian demand management, controlled economy, nationalisation, marxist analysis, welfare state expansion, education expansion and support for labour groups. Right-wing items are free-market economy, incentives, protectionism: negative, economic growth: positive, economic orthodoxy, welfare state limitation, labour groups: negative. Since the Manifesto Group includes data per election, we use the score from the last election manifesto as the party position. For the prime ministers, we use the positions of their parties. Taking party ideology as a measure for speaker ideology is unavoidable. There are no individual level estimates of the ideology of the speakers in the countries and time frame under consideration in our analyses. That said, the countries in our study are multi-party parliamentary democracies with very high levels of party discipline. For example, Sieberer [57] reports that, on average, legislators in parliamentary systems only deviate on 3 out of 100 votes. Also, in multiparty systems parties are much more cohesive ideologically than for example in a two-party system such as the United States. For these reasons party ideology is a conservative and reasonable proxy for speaker ideology.

We use standard OLS regressions. In order to evaluate the robustness of our findings, we also estimate models with fixed effects for speaker (to account for speaker-specific heterogeneity). All regression tables are listed in S1 Appendix—in the text, we focus on the main findings.

## Results

**Fig 2:**
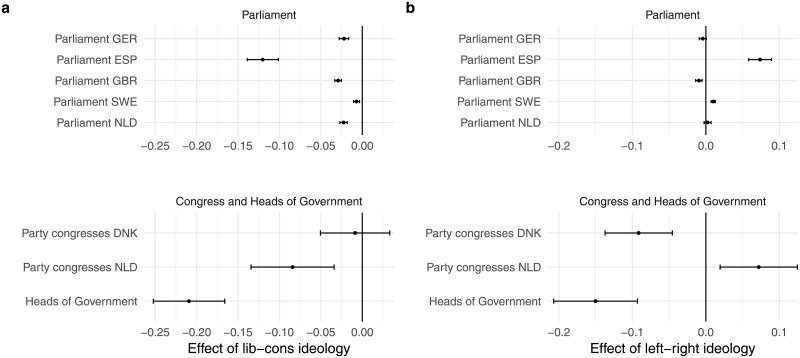
OLS regression of complexity on ideology. Plot *a* reports standardized regression coefficients for liberal-conservative ideology in eight OLS regression models (one for each dataset in the corpus). Plot *b* reports standardized regression coefficients for left-right ideology. The lines represent the 95% confidence intervals of the coefficients.

Fig 2a shows the OLS standardized regression coefficients for the effect of liberal-conservative ideology on speech complexity in each analysis. In the eight OLS regressions—one for each of the five parliamentary corpora, the two congress speeches corpora and the heads of government corpus—we find a significant result in the expected negative direction in seven cases. Only in the case of the party congress speeches in Denmark, we find an insignificant effect for liberal-conservative ideology. A likely explanation is the strong correlation between liberal-conservative ideology and economic left-right ideology (*r* = 0.80) in Denmark. Omitting that variable indeed returns a significant, negative effect for liberal-conservative ideology. A negative effect indicates that the more conservative a party, the lower the linguistic complexity of speeches of politicians from that party. We find the strongest relationship between ideology and speech complexity for heads of government: a one-standard deviation change in conservatism is estimated to decrease speech complexity by a little over 0.2 standard deviations. The effect sizes for ideology in the other corpora are more modest and vary between 0.02 (Germany) and 0.12 (Spain) standard deviations. These effect sizes are thus generally small. But this is in line with the political psychology literature that studies the association between ideology language use of politicians and other elites [9, 10, 12]. Fig 2a thus provides consistent evidence that the link between ideology and language complexity exists across countries; differences in linguistic complexity between liberals and conservatives transcend beyond the Anglo-Saxon world, despite language differences.

Fig 2b displays the results for left-right economic ideology. The results are mixed. In fact, three of the eight coefficients are positive instead of negative. Moreover, two of the eight coefficients are not statistically significant. This pattern shows that the results are inconsistent. In sum, economic left-right ideology does not systematically relate to linguistic complexity.

**Fig 3:**
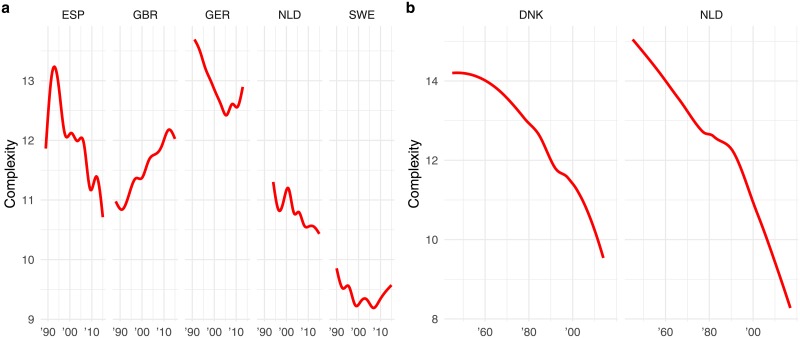
OLS regression of complexity on time. Plots *a* an *b* display loess regression lines of average speech complexity over time in the parliamentary speeches and congress speeches respectively. These are local estimates of the effect of year on complexity.

Fig 3a and 3b plot the time trends of language complexity for parliamentary speeches and party congress speeches. The party congress speeches in the Netherlands and Denmark show a steep decline in linguistic complexity over time (1945-2015). Throughout this period, complexity of party congress speeches changed from a Flesch Kincaid grade level score of approximately 16 to approximately 7. In Denmark, we observe a similar pattern, where the average Flesch Kincaid grade level score changes from approximately 14 to approximately 9. The parliamentary data by and large confirm this trend, although the time frame is more limited (1990-2015) and absolute changes are smaller: despite a few local upticks (e.g., Spain between 1990 and 1995, Germany around 2010, and the Netherlands in the early 2000s), the overall trend in speech complexity is downward. The only exception is the House of Commons (UK) where speech complexity appears to be increasing over time.

**Fig 4:**
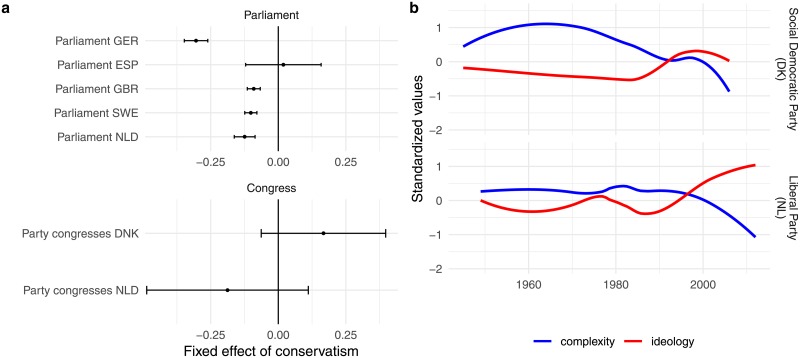
Regression of complexity on ideology with fixed effects for party. Plot *a* reports regression coefficients for liberal conservative ideology in 7 regression models with fixed effects for party (one for each dataset in the corpus with the exception of the prime minister speeches). Plot *b* displays loess regression lines of average speech complexity over time for the Danish social democratic party and the Dutch liberal party, as well as their ideological position.

Our analyses so far have picked up on ideological differences between parties, as well as the effect of ideological change within parties. In order to isolate the latter effect, we also estimate models with fixed effects for parties, zooming in on within-party variation alone. Fig 4a reports the effects of this analysis. In four out of seven corpora we find a significant, negative effect of liberal-conservative ideology on linguistic complexity. This means that when a party becomes more conservative on cultural issues (i.e., for example if they become more anti-immigrant), the linguistic complexity of their speeches decreases. We do not find any evidence for such a general pattern in Denmark and the Netherlands (congress speeches) or in Spain (parliamentary speeches). However, we do find interesting over-time variation in both the Netherlands and Denmark for specific parties. As an illustration, Fig 4b displays loess regression lines of average speech complexity over time for the Danish social democratic party and the Dutch liberal party, as well as their ideological position. For both parties, it appears that, as they become more conservative over time, they start using less complex language.

## Conclusion

This paper investigated whether conservatives—compared to liberals—use less complex language across countries, like they do in the US and the UK [2–4]. Based on our analysis of 381,609 speeches of Prime Ministers, Members of Parliament and party officials, our conclusion is that conservatives do indeed use less complex language than liberals. In seven out of eight corpora, we found a significant negative relationship between liberal-conservative ideology and speech complexity in the expected direction, and these results by and large remain in tact when we account for unobserved heterogeneity among parties by using party fixed effects. The relationship between economic left-right ideology and speech complexity, however, is much less clear. Left-wing MPs in the UK, left-wing Prime Ministers, and left-wing Danish party officials use more complex language than their right-wing counterparts, whereas in the Spanish Congresso, Swedish Riksdag and in Dutch party congresses this pattern appears to be reversed. Furthermore, we found evidence that linguistic patterns are dynamic. Parties that become more conservative, also use less complex language. Generally, we find that political language becomes less complex over time and is not systematically related to the government-opposition status of the speaker (see S1 Appendix).

Our findings offer considerable support to the claim that language conservative politicians use less complex language than liberal politicians. We replicate the American findings across different countries, time periods, and audiences, ruling out the possibility that differences in linguistic complexity among liberals and conservatives just happen to exist in set of American Senators and UK members of Parliament [2, 3, 8]. Even in complex, multidimensional European party spaces, liberal-conservative ideology is related to linguistic complexity.

Do these differences between liberals and conservatives emerge because of personality differences between these politicians? Survey research shows that Conservative MPs score higher on conscientious and lower on openness to experience than liberal or left-wing MPs [58–60]. These personality traits are associated with preferences for linguistic complexity. However, it is also possible that politicians strategically use simpler or more complex language to appeal to constituencies with distinct personality profiles and associated preferences for linguistic complexity. According to Caprara and Zimbardo [61, p. 584] a crucial skill for politicians is to learn to “speak the language of personality by identifying and conveying those individual characteristics that are most appealing at a certain time to a particular constituency”. Persuasive messages should resonate with the personality of the receiver [62]. Audience members with low need for closure and high openness to experience prefer more complex messages, and these tend to be delivered by liberal politicians. Regardless of whether it is personality or strategy, the results presented in this paper point to a more general problem in increasingly polarized democratic societies [63]: how can we find common ground, if largely irrelevant factors such as linguistic complexity can influence the public’s response.

## Supporting information

S1 Appendix Additional models and results. The Appendix contains (A) descriptive statistics for all text corpora, (B) regression results for government-opposition status, and (C) OLS and fixed effects regression tables for the models presented in this paper.
(PDF)
