# Expression of Concern: Liberals lecture, conservatives communicate: Analyzing complexity and ideology in 381,609 political speeches

**DOI:** 10.1371/journal.pone.0277860

**Published:** 2022-11-14

**Authors:** The PLOS ONE Editors

After publication of this article [1], concerns were raised about the methods and conclusions. *PLOS ONE* reassessed the article with input from members of the journal’s Editorial Board who have expertise in linguistics and social psychology research.

The Academic Editors with expertise in linguistics research advised that the Flesch-Kincaid scoring method used in the study does not meet community standards for linguistic analysis, and that this method was not appropriate or sufficient to address this study’s aims and support conclusions about linguistic complexity. An Academic Editor also raised that SPEAKER should have been modelled as a random effect rather than a fixed effect in the statistical analyses, and that instead of ordinary least squares (OLS) regression with SPEAKER as a fixed effect, the study should have used a generalized linear additive mixed-effects model with curvature for time and random intercepts for speakers and transcribers.

PLOS obtained a different perspective on the article’s scientific validity and reliability from a social psychology expert. This expert advised that the article reports a valid test of the reported hypothesis, as in their view, the method used was sufficient to assess relative differences between the complexity of communication used by liberals vs. conservatives.

The authors responded to the concerns by providing additional information, comments, and analyses. These are reported in the following section of this notice, and the materials for the reported analyses are available at https://dataverse.harvard.edu/dataset.xhtml?persistentId=doi:10.7910/DVN/S4IZ8K.

A linguistics expert advised that the additional analyses reported below suffice to lend support for the reliability of the study’s results. However, they also advised that the R-values obtained in the validation analyses (in the interval of [0.59, 0.76], corresponding to R-squared values of 34.81–57.8%) are not indicative of a robust validation outcome, and that the concerns about using the Flesch-Kincaid method remain, even considering the new analyses: better methods and tools were available and should have been used given the study’s objectives.

Regarding the statistical analysis concerns, the Academic Editor disagreed with the authors’ approach of modeling SPEAKER as a fixed effect and stands by their position that SPEAKER should instead be a random effect. Nevertheless, we consider this point as satisfactorily resolved since the authors reanalyzed the data using SPEAKER as a random effect, discussed the results obtained using the two methods, and found the main findings to be upheld using either approach.

With the added content (including modified conclusions, see section 4 below), and based on expert input received, the *PLOS ONE* Editors concluded that (a) the article’s conclusions are supported but the robustness of the conclusions is in question, (b) the study design in [1] did not meet community standards as is required by the journal’s third publication criterion, and (c) the methodological issues were not fully resolved by the post hoc analyses.

*PLOS ONE* issues this Expression of Concern to notify readers of the concerns about the methods and the robustness of the conclusions, and to provide readers the additional information and analyses reported below.

***Information and analyses provided by the authors***

## 1. Validity of the Flesch-Kincaid (F-K) grade score to assess complexity, including for source data other than written texts, and for texts not in the English language

In our paper, we apply the Flesch-Kincaid measure to written speeches across time, contexts, speakers, and topics, casting as wide a net as possible to examine the relationship between linguistic complexity and political ideology. After publication of our paper [1], criticisms were raised about the validity of Flesch-Kincaid more generally, and the way we used the measure more specifically. In response to those, we provide here a discussion of the construct, convergent, concurrent, and predictive validity of Flesch-Kincaid using existing research and additional analyses we conducted to this end. We also discuss applying Flesch-Kincaid to the languages other than English in our dataset and to spoken versus written text.

### Construct validity of Flesch-Kincaid

The Flesch-Kincaid measure was developed as a measure of *readability* of written text. It was originally linked to the US school system, indicating at what school grade pupils would be able comprehend a text. The US government has widely adopted this measure as a way of evaluating the comprehensibility of instructions (in schools, in the army but also for medicine). In scientific research, “traditional readability formulas are still in in widespread use today” [2]. Wang and colleagues found that the Flesch-Kincaid grade level was the most commonly used readability formula in medicine [3]. In political science, the measure is also widely used to examine political language [4–14].

Flesch-Kincaid is criticized for having weak construct validity, because it is “not based on theories of reading or comprehension but rather rely on statistical correlations to develop predictive power” [2]. It also does not “take into consideration relationships between elements in the text” and the impact that particular styles, vocabulary or grammar may have [2,15]. Yet, it has also been argued that to pick up on these latter components, different definitions of complexity need to be developed–such as a distinction between semantic and syntactic complexity [16]. Indeed, Flesch-Kincaid has limitations when interpreted as a *direct* measure of reading comprehension [17] since it is solely based on syntactic and lexical features (structural elements of a text) and not on semantic features (variation in word use and textual content).

In sum, Flesch-Kincaid is “based on only two levels of linguistic features (i.e., lexical and syntactic), and these features are, at best, proxies of the features recognized as important during linguistic processing” [2,15]. Yet, the same authors do acknowledge that “these formulas do give a rough estimate of difficulty” [2]. Thus, construct validity of Flesch-Kincaid as a measure of linguistic complexity is still debated. We contend that to validate Flesch-Kincaid we also need to examine its convergent validity (how does Flesch-Kincaid compare with other measures of linguistic complexity?) and its concurrent validity (to what degree does Flesch-Kincaid scores of political speeches correlate with comprehension among members of the audience?)

### Convergent validity of Flesch-Kincaid

In assessing the convergent validity of Flesch-Kincaid, we first discuss the existing literature on the matter, before turning to our own efforts at validation. It is important to note that even strong advocates of using alternative measures than Flesch-Kincaid report moderate correlations between text processing judgments of readers and Flesch-Kincaid (r = 0.39) [2,15]. These authors provide a measure that outperforms Flesch-Kincaid, but they still maintain that Flesch-Kincaid provides “a rough estimate of difficulty” [2]. Similar results can be found in validation efforts in political science and medicine. Benoit et al. [8] had 19,430 text snippets from US State of the Union addresses (i.e. written-to-be-spoken, comparable to other political speeches) coded by humans. Each time coders were given two text snippets and asked to indicate which one was the easiest to comprehend. Benoit et al. then predicted these human validation scores using the F-K scores of the text snippets. The Flesch-Kincaid scores correctly predicted 72% of the cases. Moreover, Benoit et al. also show that adding more syntactic and semantic features to the analysis only marginally improves predictions of how complex a text is. A recent pre-registered, survey experiment similarly found that syntactic components of Flesch-Kincaid predict human-coded sophistication of political text in Germany [9]. Furthermore, Grabeel and co-authors had human coders score the linguistic complexity of 148 medical brochures. To this end, they used the so-called Simple Measure of Gobbledygook (SMOG) and contrasted it with Flesch Kincaid. The authors only produced a scatterplot, which suggested that the correlation between SMOG and F-K is around .8 [18]. In combination, these findings evidence that Flesch-Kincaid measures correlate meaningfully with other, human-coded measures of textual complexity.

In order to validate Flesch-Kincaid further, we first went back to our data to examine how it correlates with 4 other measures of syntactic complexity used in linguistics (see e.g., [19,20]). [Replication materials for all analyses in this notice are available here: https://dataverse.harvard.edu/dataset.xhtml?persistentId=doi:10.7910/DVN/S4IZ8K] Because estimating these measures would take multiple days in our corpus of 381,609 speeches, we took random samples of 4000 parliamentary speeches in 4 countries, as well as Congressional speech data in the Netherlands, and Prime Minister speeches in the English language. Together these corpora represent 4 languages (English, Spanish, German and Dutch) and a large majority of speeches in our article. We used the spacyr library [21] to process these corpora using language-specific parsers in the Python spaCy environment. Because the spaCy library did not include Danish and Swedish language parsers, we did not include those corpora in this analysis. Using the textplex library in R [22] we then obtained four readability and syntactic complexity measures that have been validated on political text [e.g., 23,24,25]: the automated readability index (ARI), average sentence length, as well as syntactic depth and syntactic dependency. These are both measures of the average number of links between the top node and the terminal node when sentences are modeled in a tree-like structure. We then standardized these measures and took their average to obtain a standardized syntactic complexity scale.

S1 File displays the correlations between F-K and this syntactic complexity scale of 4 measures in the 6 corpora. These correlations are high [26], varying between 0.59 in the UK and 0.76 in the Dutch Congress speeches. S2 File displays the correlations between Flesch-Kincaid and a scaled measure of the two language-independent readability scores: ARI, which only includes on the number of characters and words in a sentence (other than Flesch-Kincaid which relies on syllables) and sentence length. These correlations are very high ranging from r = 0.82 in the British House of Commons to r = 0.97 in Dutch Congress speeches.

These high correlations between the F-K measure and other measures of complexity provide evidence of the convergent validity of the Flesch-Kincaid measure: across languages and speeches, the measure we employed in our paper correlate very highly with other measures of syntactic complexity.

In a second step, we re-analyzed the statistical models presented in Fig 2 of our article [1], but this time with two versions of the syntactic complexity scale: one that contains only the average readability index and average sentence length (“2 indicators of syntactic complexity”) and one that contains all four measures (“four indicators of syntactic complexity”). S3 File displays the results of this exercise. In 5 out of 6 corpora we replicate, with other measures of syntactic complexity, our finding that progressive politicians use more complex language than conservative politicians, evidenced by a negative regression coefficient of liberal-conservative ideology on syntactic complexity. The only exception is the House of Commons, a result that is in part driven by the fact that—in comparison to the other corpora for which we have speakers from many different parties and with a large variety in ideology scores—speakers from just two parties (Labour and the Conservatives) dominate the discourse in the House of Commons.

To conclude, the evidence we document here offers convergent validation of Flesch-Kincaid as a measure of complexity as it correlates highly with more recent measures of syntactic complexity. Moreover, using these other, more recent, measures of syntactic complexity as a dependent variable in our regression models leads to similar conclusions as those we draw in our paper.

### Concurrent validity of Flesch-Kincaid

To what degree do these F-K scores correlate with comprehension among members of the audience? In what follows, we bring new to on this question, supporting our viewpoint that Flesch-Kincaid scores are *correlated* with comprehension among audience members.

We examined how Flesch-Kincaid correlates with intelligibility across languages. We tested this for five of the six main languages (Dutch, English, German, Spanish and Swedish) in our study, representing 99.9% of our data. We test whether Flesch-Kincaid scores correlate with how audience members perceive the complexity of text. We selected 20 text fragments per language (mostly consisting of one sentence, some of two sentences) with varying Flesch-Kincaid scores from different political speeches in our original dataset. We asked respondents to code how complex they found these fragments on a scale from 0 (not complex at all) to 100 (very complex). We also recorded 45 audio fragments (15 per language) with varying Flesch-Kincaid scores in Dutch, English and German respectively, and again asked respondents to code how complex they found the fragment that was read read to them. These audio fragments were recorded by native speakers.

The study was approved by the Ethics Review Board of the University of Amsterdam (#2021-CS-13229). We launched the study in the subject-pool of participants maintained by the Behavioural Science Lab of the Faculty of Social and Behavioural Sciences at the University of Amsterdam. We recruited native speakers of the relevant languages only. In return for their participation, respondents received credits that they need for the completion of their Bachelor’s degree. Because few students in the pool are Swedish, we additionally recruited students from a contact at a Swedish university. In total, 152 participants coded 4,683 text and audio fragments.

Table 1 shows the correlations between the Flesch-Kincaid scores of the sentences in the validation tasks with the mean human coding scores. These correlations are very high (r > .85) in 5 out of 8 cases and high in the other 3 cases (r > .7). Our results thus show that Flesch-Kincaid scores correlate strongly with perceived complexity across the five languages.

**Table 1:** Correlations between Flesch-Kincaid of written and spoken text fragments with human coded complexity.

Language	Mode	Correlation with Flesch Kincaid	N respondents/ sentences coded
Dutch	audio	**0.886**	**64 / 864**
Dutch	text	**0.890**	**64 / 1180**
English	audio	**0.779**	**59 / 954**
English	text	**0.929**	**59 / 960**
German	audio	**0.946**	**9 / 135**
German	text	**0.887**	**9 / 180**
Spanish	text	**0.729**	**11 / 220**
Swedish	text	**0.756**	**9 / 180**

Regarding the difference between audio and text: In the Dutch case, the correlations of the audio and text fragments are almost identical (difference of .004). In English, the correlation with the text fragments is somewhat higher (difference of 0.15), and in German the correlation with the audio fragments is somewhat higher (difference of 0.059).

In sum, our findings here support the concurrent validity of the Flesch-Kincaid measure, across language, and types of communication. This is in line with other work political science. For example, individuals are better able to locate parties’ ideological positions if they have less complex election manifestos (lower Flesch-Kincaid scores) [10]. Voters are less likely to answer ballot questions that score low on readability [11]; when asked low readability survey questions, respondents are more likely to answer ‘don’t know’ or to adhere to heuristics in their responses [12, 13]; low readability in political speeches correlates positively with other indicators of comprehension such as low familiarity of words and high abstractness [14].

### Validation across languages

The Flesch-Kincaid score has been developed to assess readability of English text, but has been adapted to other languages, e.g. Lesbarkeitsindex (LIX) for German and the Flesch-Douma index for Dutch. Both the Lesbarkeitsindex and Flesch Douma correlate very strongly (r = 0.99) with the original Flesch-Kincaid method applied to German and Dutch in our data [1]. Also, the correlations between human coding and the Flesch-Kincaid measure presented in Table 1 is as high for German, Dutch, and Spanish, as it is for English. On the basis of this evidence, the Flesch-Kincaid measure has a similar degree of concurrent and convergent validity across languages.

### Validation for spoken language vs written language

Spoken language is different from written language [27,28] and this raises important questions about what Flesch-Kincaid measures when applied to transcripts of speeches. Political speeches are often pre-written and thoroughly prepared, resembling written language more closely than day-to-day conversation. Two out of three datasets that our paper relies on consist only of pre-written speeches (the prime minister speeches that are part of EUSpeech [29], and the party congress speeches [30]); one dataset consists of transcribed speeches, some of which are pre-written while others are not (Parlspeech [31]). We note that our results are consistent across the data sources that contain either spoken or written language. In addition, in Table 1 we report few to no differences between the human judgments of complexity between the spoken and the written language, across all languages. In the next section we show evidence that the transcription process is very unlikely to have impacted the findings in our paper.

### Conclusion

In sum, our validations show that Flesch-Kincaid scores correspond closely to human complexity assessments of written *and* spoken political statements. We have also shown that the conclusions we draw in our paper would have been similar had we used other measures of syntactic complexity. At this point, it is important to underscore that we do not make the claim in the paper that language of low or high complexity is inherently more or less understandable (see our discussion of construct validity). Rather, what we are interested in is how such language is perceived by the public. Our survey data shows that—across 5 languages—language of higher complexity as measured by F-K is indeed perceived to be more complex by native speakers. And this result is similar for evaluations of both written and spoken text.

## 2. Statistical analyses

Concerns were raised about the type of model applied in the statistical analyses, the designation of SPEAKER as a fixed effect rather than a random effect in the regression analyses, and whether/how transcriber effects were addressed.

### The designation of SPEAKER as a fixed effect rather than a random effect in the regression analyses

In our article, we use fixed effects for individuals (dummy variables for speakers) to account for unobserved heterogeneity among speakers. The variation in linguistic complexity that we are left with is what we regress on our party-based measure of ideology. The interpretation of this fixed effects model is as follows: for a given member of a party, as their party becomes more liberal or conservative how does this impact the complexity of their language? Or to put it differently, as a party becomes more (or less) conservative over time, what is the impact on the complexity of speeches of the “average” party member? We believe that this offers additional insight in the relationship between ideology and language complexity beyond our main statistical model by focusing on over-time variation within parties.

To examine if these results are any different for a random intercept specification, we have re-analyzed the results in Fig 4 of our article [1] with a random intercept for speaker instead of fixed effects. S4 File displays the coefficients for the fixed effects model (that is, Fig 4 in [1]) on the left panel and the random effects coefficients in the right panel. The estimated coefficients for liberal-conservative ideology in these random-intercept models are largely identical to the coefficients of liberal-conservative ideology in the fixed effects model presented in Fig 4 in [1]. The one exception is the congress speeches analysis in the Netherlands. While in the fixed effects model, liberal-conservative ideology had a negative but not statistically significant effect on linguistic complexity, in the random intercept model the coefficient of liberal-conservative ideology is still negative but also statistically significant, in line with the other findings. The results in S4 File show that we arrive at identical substantive conclusions, regardless of whether we use fixed or random effects for speakers.

To summarize, the results we present here give no reason to conclude that the results presented in our paper are conditional upon the decision to conclude fixed effects or random effects.

### Whether/how transcriber effects were addressed

It was also posited that there is a risk that transcriber effects can have impacted the regression results. As far as we can tell, this can imply two different claims: 1) transcribers will transcribe speeches from liberal speakers systematically differently than speeches from conservative speakers, 2) random transcription errors will have impacted the results. In what follows we argue that neither of these scenarios is likely, relying on evidence from inquiries with teams of transcribers in 5 parliaments as well as simulations in which we mimic scenarios in which transcribers make more and more random transcription errors to see how these may impact our regression results.

We contacted transcriber teams in the 5 parliaments in our dataset—the Dutch Tweede Kamer, the German Bundestag, the Swedish Riksdagen, the British House of Commons and the Spanish Congreso—and asked them how spoken speeches get transcribed—correspondence can be made available. Table 2 summarizes the workflow of the transcriber teams in these 5 parliaments.

**Table 2:** Procedures of transcription in different parliaments.

Country	Stated Goal	Procedure	Quality Control
**Germany: Bundestag**	High consistency between different stenographers, a transcription ‘aus einem Guss’.	16 stenographers transcribe the speeches, rotating every five minutes.	Yes: eight individuals check the protocol for completeness, accuracy, and consistency.
**United Kingdom: House of Commons**	A “substantially verbatim” report of what is said in Parliament	Approximately 50 editorial members work to transcribe proceedings. Assignments to debates are essentially random, depending on how many debates take place and how long they go for.	Yes: All Hansard transcribers undergo extensive training for six months. Proceedings are captured by digital audio from which the edited verbatim accounts are reported. They are then checked by subeditors before publication online. They are subsequently proofread, corrected if necessary and statistics on numbers of columns and errors are collated.
**Sweden: Riksdag**	“In as far as it is possible, the record should be a verbatim reproduction of what has been said during the meetings”	18 stenographers work here during the day, 12 in the evening. Stenographers rotate every ten minutes. After ten minutes in the chamber, they have two hours to transcribe the recording of “their” ten minutes.	Yes: There are experienced transcribers (so called “Protokollsgranskare”) whose main task is checking that transcriptions are correct.
**NL: Tweede Kamer**	The Handelingen are a “complete and as literal as possible” report of what is said in Parliament	During plenary debates up to more than 30 transcribers work in short shifts	Yes: Transcriptions are checked by a team of stenographers. Speakers have ‘correctierecht’–the right to correct the transcriptions if they find it doesn’t reflect what they said.
**Spain: Congreso**	“Producing a public, official document that is readable and understandable both today and 50 years from now”	Team of 33, with 17 baseline stenographers, who work in 5–10 minutes shifts.	Yes: Transcriptions are checked by other stenographers. In case of discrepancies, the whole team assesses the issue.

**Note:** the information in this table was collated by consulting the websites of the 5 parliaments and inquiring directly with the transcriber teams in each parliament.

In general, transcribers work in teams of about a dozen transcribers responsible for one legislative debate. Throughout a debate, individual transcribers are sent to the floor for a short period of time (typically 5 to 10 minutes) during which they transcribe the ongoing debate. After they have been to the floor, transcribers then work out their transcriptions. The quality of the worked-out transcription is checked on a team basis in order to guarantee quality, coherence, and comparability among the various transcribers. For example, in the Spanish Congreso, the transcription unit at the lower house is composed of a 33-strong team. 17 of them are baseline stenographers who work in 5–10 minutes shifts in both the plenary and specific commission sessions. In large, plenary sessions, they operate in teams of 12, supervised by 6 other stenographers who revise their transcriptions.

The workflows in these parliaments make it extremely unlikely that there are systematic individual transcriber effects for speakers of a particular ideology. First, the assignment of transcribers to the floor is uncorrelated with the ideology of who happens to be on the floor speaking. It is not the case that transcribers are assigned to particular speakers. Second, all 5 parliaments have built into their transcription procedures extensive, multi-layered quality control. Put differently, transcription is a collective endeavor. Our inquiry with the 5 parliaments provided no indication that indeed transcriber effects are a meaningful confounding variable. To conclude, we find no evidence that transcriber effects are a confounding factor in our study.

What about random transcription errors? As explained in the previous paragraph, we have no direct way of picking up transcription errors in our data. Therefore, we chose a different procedure. If there were transcription errors, then our Flesch-Kincaid measure would contain a certain degree of noise. It is an empirical question whether our results hold if we add random noise to our Flesch-Kincaid measures, mimicking a scenario in which transcribers randomly make punctuation errors, or otherwise alter the measured complexity of the transcribed speeches.

To investigate whether transcription errors would affect our results we ran 1,800 simulations. For each of the 5 parliaments (95% of data in our sample) we randomly generated 100 variables that have a correlation to the original Flesch-Kincaid score of 0.25 (low), 0.5 (modest) or 0.75 (high). We subsequently re-ran the analysis with these simulated complexity scores. From these analyses we extracted the beta coefficient of the liberalism-conservatism variable (y-axis in S5 File) and the associated p-value (x-axis in S5 File). S5 File shows the beta and associated p-value of liberalism-conservatism of each simulation. The red line demarcates the difference between p-values smaller or larger than .05. In most cases, there is a collection of dots to the left of the red line. This means that in most, if not all, cases we replicated the original finding of a statistically significant negative effect. For each panel, we note the percentage of cases in which we replicated the analysis.

In the bottom panel we show the simulations in which we use a dependent variable with a correlation of r = 0.75 to the original Flesch-Kincaid score. In 98% of the simulations, we replicate the original result. Actually, in 4 out of 5 countries, we replicate the result in all 100 simulations. Only in Sweden, this is slightly lower (90%), but we still always find a negative effect.

In sum, if we conservatively assume the lowest correlation from the human validation task, we still replicate the original result in 98% of the cases using 5 entirely different samples. If we assume lower correlations (e.g. r = 0.5 or r = 0.25) with the original dependent variable, then we are simulating a scenario where there are a lot of transcription errors. Even in these scenarios, we are still likely to find a statistically significant, negative relationship. Assuming r = 0.5, only the Swedish results are somewhat weaker. If we assume the very low correlation of r = 0.25 we still find a negative and significant relationship in 62.4% of the cases (this is the average of the percentages of the countries in the top row (45%, 72%, 86%, 15% and 94%). In the pooled sample (i.e. all samples combined), we replicate the original result in 100% of the cases, even if the correlation is low. In sum, we would have to make extreme assumptions (e.g r = 0) about the relationship between Flesch-Kincaid and complexity for our findings to collapse. Flesch-Kincaid is a noisy measure, but the level of noise that we identified in the human validation task still produces extremely reliable results.

To conclude, these simulations demonstrate that random transcription errors are very unlikely to lead to different substantive conclusions.

## 3. Additional limitations of the study design

The dataset was comprised of a heterogeneous sample, including source text of different types, languages, and transcribed text. Furthermore, for transcribed texts, punctuation was applied differently across the dataset as per the transcriber’s preference.

In our paper, we apply the Flesch-Kincaid measure to transcripts of speeches across time, contexts, speakers and topics but we present our results for each language and political institution separately. We discuss our rationale for using F-K in our manuscript and rely on other validations [e.g., 4–14]. But we did not provide original validations to support the use of F-K across languages and institutions. We offered these validations in this notice.

In particular, we have (a) cross-validated the Flesch-Kincaid measure with a range of other measures of syntactic complexity (see S1, S2, S3 Files), (b) conducted a qualitative inquiry of the transcription process of 5 parliaments (see Table 2) and simulated possible effects of random transcriptions errors (see S5 File), (c) validated Flesch-Kincaid on both spoken and written language through an original survey among 152 respondents across 5 languages (see Table 1) and (d) compared the robustness of our fixed effects regression results against a random effects specification (see S4 File). Our analyses show that the results we present in our article are valid and reliable, that transcriber effects are very unlikely to be an issue, and that an alternative statistical modelling strategy does not change the conclusions we draw in our article.

## 4. Conclusions

The conclusions reported in [1] were overstated in light of the study’s limitations. The conclusions are revised to: Our results suggest that speakers from culturally liberal parties use more complex language than speakers from culturally conservative parties, and that economic left-right differences are not systematically linked to linguistic complexity. Further studies—for example including subgroup analyses and additional complexity measures—are needed to confirm and verify these findings.

Flesch-Kincaid has limitations when interpreted as a *direct* measure of comprehension [17] since it is solely based on syntactic and lexical features and not on semantic features. Despite this limitation, in this notice we have offered extensive evidence that we can use Flesch-Kincaid to compare perceived complexity of language in large-scale corpora of political speeches.

## Supporting information

S1 File Correlations of F-K and a scale of 4 measures of syntactic complexity in 6 corpora. (PNG)

S2 File Correlations of F-K and a scale of 2 measures of syntactic complexity in 6 corpora. (PNG)

S3 File Estimated regression coefficients of liberal conservative ideology on syntactic complexity. (PNG)

S4 File Effect of conservatism with fixed effects and random intercepts for speakers. (PNG)

S5 File Simulated effects with random noise added to F-K, 100 simulations per facet. (JPG)
