# Supplementary material for: Liberals lecture, conservatives communicate: Analyzing complexity and ideology in 381,609 political speeches
Source: PLoS One. 2019 Feb 6;14(2):e0208450. doi: 10.1371/journal.pone.0208450 (PMC6364865; doi:10.1371/journal.pone.0208450)
Supplement: S1 Appendix — The Appendix contains (A) descriptive statistics for all text corpora, (B) regression results for government-opposition status, and (C) OLS and fixed effects regression tables for the models presented in this paper. (PDF) [file pone.0208450.s001.pdf]

Appendix for: ***Liberals Lecture, Conservatives Communicate: Analyzing Complexity and Ideology in 381,609 Political Speeches***

Martijn Schoonvelde[1*], Anna Brosius[2], Gijs Schumacher[3], Bert N. Bakker[2]

1 School of Politics and International Relations, University College Dublin, Dublin, Ireland
2 Amsterdam School of Communication Research, University of Amsterdam, Amsterdam, Noord-Holland, the Netherlands
3 Department of Political Science, University of Amsterdam, Amsterdam, Noord-Holland, the Netherlands

* Corresponding author. Email: mschoonvelde@gmail.com

A Descriptive statistics in all eight corpora

Table A.1: Party congresses DNK

Statistic	N	Mean	St. Dev.	Min	Pctl(25)	Pctl(75)	Max
Complexity	528	−0.046	0.771	−1.396	−0.513	0.315	6.435
Left-Right	528	−0.222	1.624	−3.925	−1.156	1.312	3.104
Progressive-Conservative	528	0.405	1.688	−2.938	−0.672	1.120	6.037
Year	528	44.670	15.520	0	33.8	57	69
In government	528	0.347	0.476	0	0	1	1

Table A.2: Party congresses NLD

Statistic	N	Mean	St. Dev.	Min	Pctl(25)	Pctl(75)	Max
Complexity	659	−0.057	0.697	−1.493	−0.583	0.418	2.486
Left-Right	659	−0.080	0.889	−2.011	−0.721	0.633	1.807
Progressive-Conservative	659	−0.582	0.905	−2.543	−1.140	−0.133	2.622
Year	659	39.420	20.514	1	22	58	72
In government	659	0.584	0.493	0	0	1	1

Table A.3: Parliament GER

Statistic	N	Mean	St. Dev.	Min	Pctl(25)	Pctl(75)	Max
Complexity	60,763	0.150	0.488	−2.030	−0.192	0.451	6.756
Left-Right	60,763	−0.326	1.185	−2.921	−1.235	0.740	1.485
Progressive-Conservative	60,763	−0.290	0.972	−2.912	−0.889	0.189	1.336
Year	60,763	12.342	6.714	0	7	19	22
In government	60,763	0.548	0.498	0	0	1	1

Table A.4: Parliament ESP

Statistic	N	Mean	St. Dev.	Min	Pctl(25)	Pctl(75)	Max
Complexity	35,154	2.502	1.051	−1.795	1.786	3.143	9.028
Left-Right	35,154	−0.213	0.790	−2.239	−0.859	0.495	1.234
Progressive-Conservative	35,154	−0.853	0.662	−2.576	−1.397	−0.300	0.206
Year	35,154	13.212	7.264	0	7	20	26
In government	35,154	0.371	0.483	0	0	1	1

Table A.5: Parliament GBR

Statistic	N	Mean	St. Dev.	Min	Pctl(25)	Pctl(75)	Max
Complexity	161,439	−0.178	0.465	−2.716	−0.461	0.109	8.640
Left-Right	161,439	0.146	0.705	−0.841	−0.448	0.684	1.666
Progressive-Conservative	161,439	0.527	0.745	−1.124	−0.028	0.839	1.844
Year	161,439	14.014	7.718	0	7	21	27
In government	161,439	0.545	0.498	0	0	1	1

Table A.6: Parliament SWE

Statistic	N	Mean	St. Dev.	Min	Pctl(25)	Pctl(75)	Max
Complexity	72,955	−0.728	0.414	−3.088	−1.006	−0.472	2.093
Left-Right	72,955	0.152	1.364	−3.260	−0.831	0.891	3.613
Progressive-Conservative	72,955	−0.501	0.998	−5.112	−0.995	0.254	2.056
Year	72,955	13.051	7.294	0	7	19	25
In government	72,955	0.391	0.488	0	0	1	1

Table A.7: Parliament NLD

Statistic	N	Mean	St. Dev.	Min	Pctl(25)	Pctl(75)	Max
Complexity	48,281	−0.383	0.378	−2.109	−0.629	−0.175	4.625
Left-Right	48,281	−0.140	0.929	−3.100	−0.672	0.403	1.469
Progressive-Conservative	48,281	0.006	0.991	−2.613	−0.739	0.593	2.622
Year	48,281	11.664	6.098	0	6	17	21
In government	48,281	0.551	0.497	0	0	1	1

Table A.8: Heads of Government

Statistic	N	Mean	St. Dev.	Min	Pctl(25)	Pctl(75)	Max
Complexity	1,830	0.089	0.756	−2.210	−0.446	0.547	5.865
Left-Right	1,830	0.010	0.760	−2.356	−0.921	0.684	1.102
Progressive-Conservative	1,830	−0.079	1.005	−3.628	−0.487	0.828	3.110

B Government-opposition status

What about the impact of government-opposition on linguistic complexity? Figure B.1 shows the regression coefficient for the government-opposition status variable, which indicates the difference between government parties and opposition parties. Surprisingly, the results are rather inconsistent: Government parties use more complex language than opposition parties in Germany, Sweden and Denmark, but the pattern is reversed in the United Kingdom, and we find no relationship between government status and complexity in Spain. Furthermore, the Dutch parliamentary data indicate a positive relationship, whereas the party congress data indicate no relationship. These results show that, across countries and contexts, the relationship of being in government and language complexity is less clear-cut than in the US-American and Canadian contexts where speakers from opposition parties use less complex language than speakers from government parties (see for example Tetlock, 1981; Pancer et al., 1992).

Figure B.1: OLS regression of complexity on government status and time

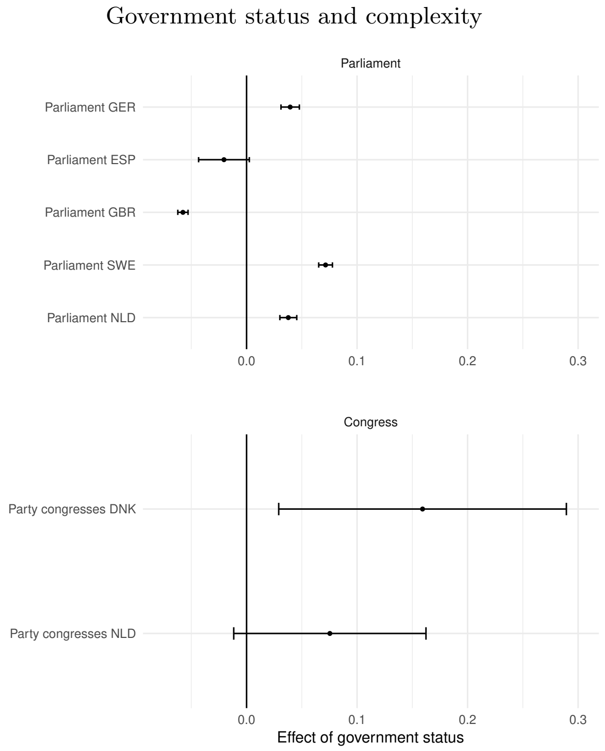

Note: This plot reports standardized regression coefficients for government-opposition status in seven OLS regression models (one for each dataset in the corpus with the prime minister speeches excluded). The bars represent the 95% confidence intervals of the coefficients.

References

Pancer, S Mark, Bruce Hunsberger, Michael W Pratt, Sylvie Boisvert and Don Roth. 1992. “Political Roles and the Complexity of Political Rhetoric.” *Political Psychology* 13(1):31–43.

Tetlock, Philip E. 1981. “Pre- to postelection shifts in presidential rhetoric: Impression management or cognitive adjustment.” *Journal of Personality and Social Psychology* 41(2):207–212.

C OLS regression models of complexity

Table C.1: OLS regression results

	DNK Congress	NLD Congress	GER Parl	ESP Parl
Left-Right	-0.091^{***}	0.072^{***}	-0.004^{*}	0.074^{***}
	(0.023)	(0.027)	(0.002)	(0.008)
Progressive-Conservative	-0.009	-0.084^{***}	-0.022^{***}	-0.120^{***}
	(0.021)	(0.026)	(0.003)	(0.010)
Year	-0.021^{***}	-0.022^{***}	-0.009^{***}	-0.020^{***}
	(0.002)	(0.001)	(0.0003)	(0.001)
In government	0.159^{**}	0.075^{*}	0.039^{***}	-0.020^{*}
	(0.066)	(0.044)	(0.004)	(0.012)
Constant	0.836^{***}	0.736^{***}	0.235^{***}	2.682^{***}
	(0.113)	(0.061)	(0.005)	(0.015)
Observations	528	659	60,763	35,154
R^2	0.181	0.481	0.022	0.026
Adjusted R^2	0.175	0.478	0.022	0.026
Residual Std. Error	0.700 (df = 523)	0.504 (df = 654)	0.483 (df = 60758)	1.037 (df = 35149)
F Statistic	28.868^{***} (df = 4; 523)	151.337^{***} (df = 4; 654)	348.687^{***} (df = 4; 60758)	235.073^{***} (df = 4; 35149)
Note:				*p<0.1; **p<0.05; ***p<0.01

	GBR Parl	SWE Parl	NLD Parl	PM
Left-Right	-0.010^{***}	0.010^{***}	0.002	-0.150^{***}
	(0.002)	(0.001)	(0.002)	(0.029)
Progressive-Conservative	-0.029^{***}	-0.007^{***}	-0.023^{***}	-0.209^{***}
	(0.002)	(0.002)	(0.002)	(0.022)
Year	0.014^{***}	-0.001^{**}	-0.007^{***}	
	(0.0001)	(0.0003)	(0.0003)	
In government	-0.058^{***}	0.071^{***}	0.038^{***}	
	(0.002)	(0.003)	(0.004)	
Constant	-0.328^{***}	-0.753^{***}	-0.326^{***}	0.074^{***}
	(0.003)	(0.005)	(0.005)	(0.016)
Observations	161,439	72,955	48,281	1,830
R^2	0.065	0.009	0.020	0.157
Adjusted R^2	0.065	0.009	0.020	0.156
Residual Std. Error	0.450 (df = 161434)	0.412 (df = 72950)	0.375 (df = 48276)	0.695 (df = 1827)
F Statistic	$2,809.898^{***}$ (df = 4; 161434)	164.229^{***} (df = 4; 72950)	246.653^{***} (df = 4; 48276)	169.938^{***} (df = 2; 1827)
Note:				*p<0.1; **p<0.05; ***p<0.01

D Speaker fixed effects regression models of complexity

Table D.1: Speaker fixed effects regression results

	DNK Congress	NLD Congress	GER Parl	ESP Parl
Left-Right	0.364**	0.141	0.019	−0.133**
	(0.154)	(0.192)	(0.020)	(0.061)
Progressive-Conservative	0.167	−0.188	−0.304***	0.019
	(0.117)	(0.152)	(0.022)	(0.071)
Year	−0.067***	−0.087***	−0.027***	−0.082***
	(0.010)	(0.005)	(0.001)	(0.003)
In government	1.210***	0.234	0.088***	0.852***
	(0.274)	(0.187)	(0.017)	(0.052)
Constant	15.104***	14.672***	13.280***	24.207***
	(0.716)	(0.304)	(0.025)	(0.210)
Party Fixed Effects?	*Yes*	*Yes*	*Yes*	*Yes*
Observations	528	659	60,763	35,154
R^2	0.239	0.500	0.027	0.064
Adjusted R^2	0.227	0.490	0.027	0.064
Residual Std. Error	2.652 (df = 519)	1.948 (df = 645)	1.885 (df = 60754)	3.980 (df = 35141)
F Statistic	20.367*** (df = 8; 519)	49.557*** (df = 13; 645)	211.974*** (df = 8; 60754)	200.657*** (df = 12; 35141)

Note: *p<0.1; **p<0.05; ***p<0.01

	GBR Parl	SWE Parl	NLD Parl
Left-Right	−0.072***	0.037***	0.0003
	(0.014)	(0.009)	(0.016)
Progressive-Conservative	−0.091***	−0.102***	−0.124***
	(0.012)	(0.011)	(0.020)
Year	0.054***	−0.005***	−0.028***
	(0.001)	(0.001)	(0.001)
In government	−0.198***	0.302***	0.038**
	(0.010)	(0.013)	(0.018)
Constant	10.866***	9.178***	11.113***
	(0.014)	(0.030)	(0.028)
Party Fixed Effects?	*Yes*	*Yes*	*Yes*
Observations	161,439	72,955	48,281
R^2	0.068	0.022	0.036
Adjusted R^2	0.068	0.022	0.035
Residual Std. Error	1.759 (df = 161430)	1.602 (df = 72942)	1.455 (df = 48264)
F Statistic	1,464.877*** (df = 8; 161430)	136.353*** (df = 12; 72942)	111.615*** (df = 16; 48264)

Note: *p<0.1; **p<0.05; ***p<0.01
