# Supplementary figures and images for: Expression of Concern: Liberals lecture, conservatives communicate: Analyzing complexity and ideology in 381,609 political speeches
Source: PLoS One. 2022 Nov 14;17(11):e0277860. doi: 10.1371/journal.pone.0277860 (PMC9662719; doi:10.1371/journal.pone.0277860)

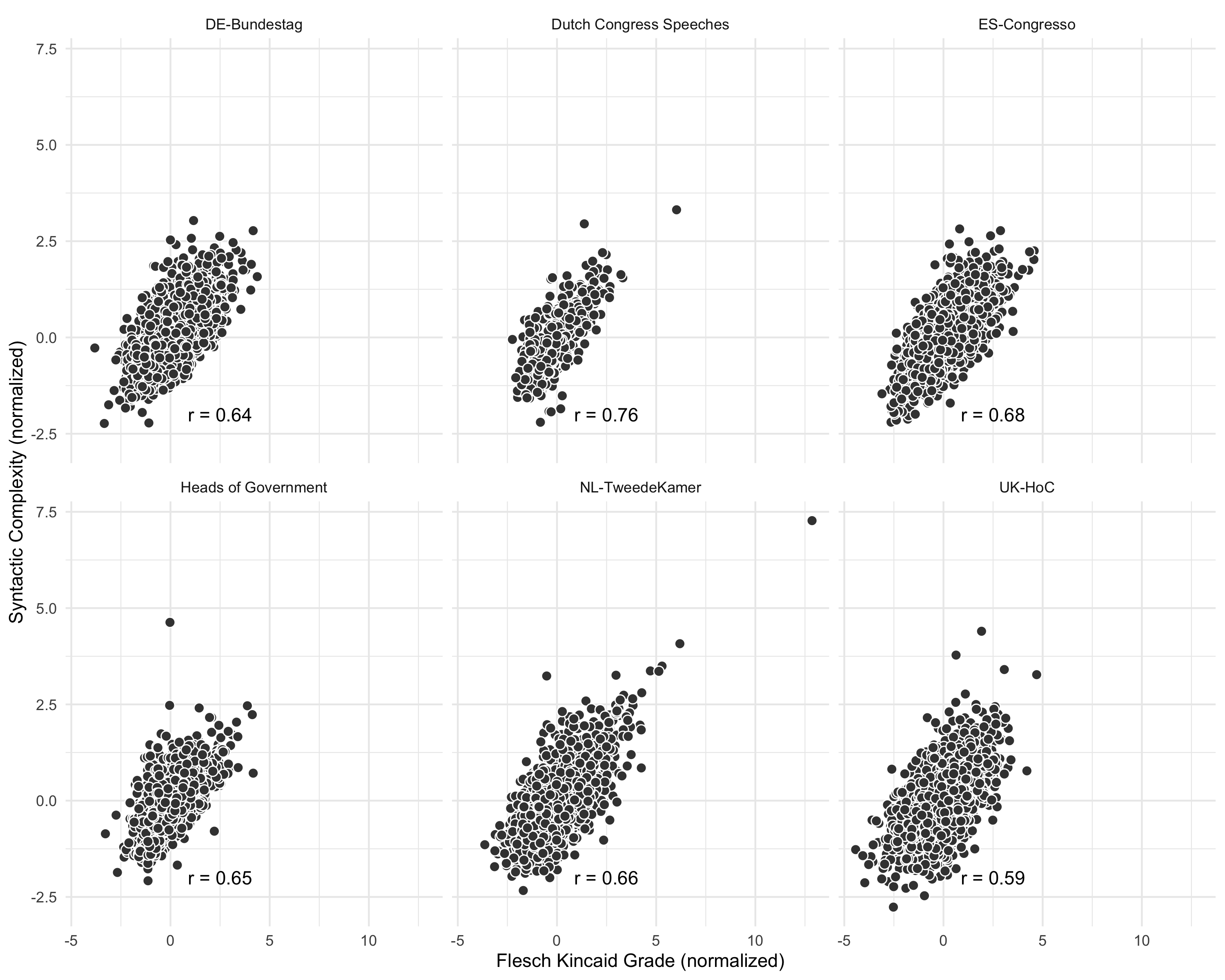

Supplement: S1 File — (PNG) [file pone.0277860.s001.png]

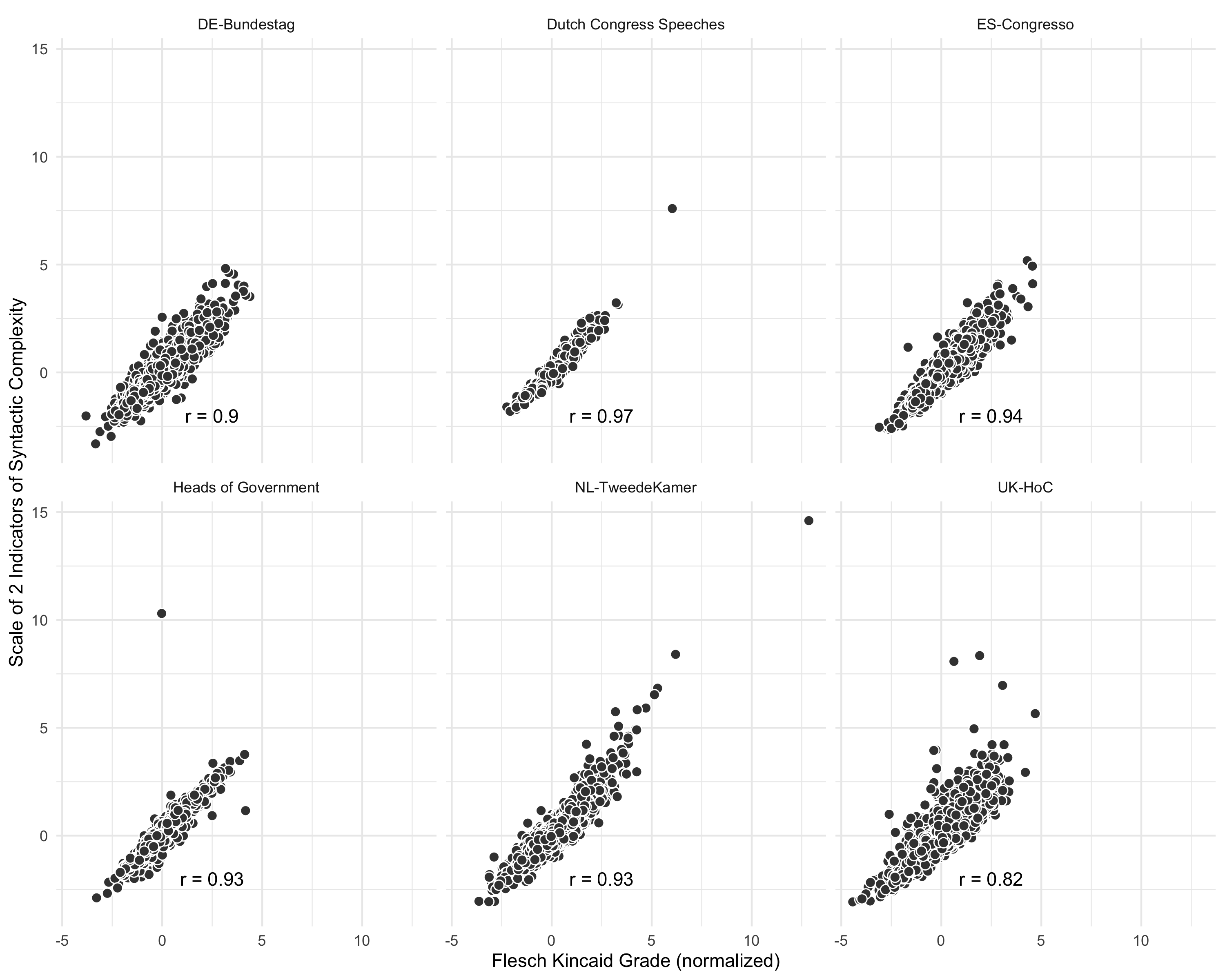

Supplement: S2 File — (PNG) [file pone.0277860.s002.png]

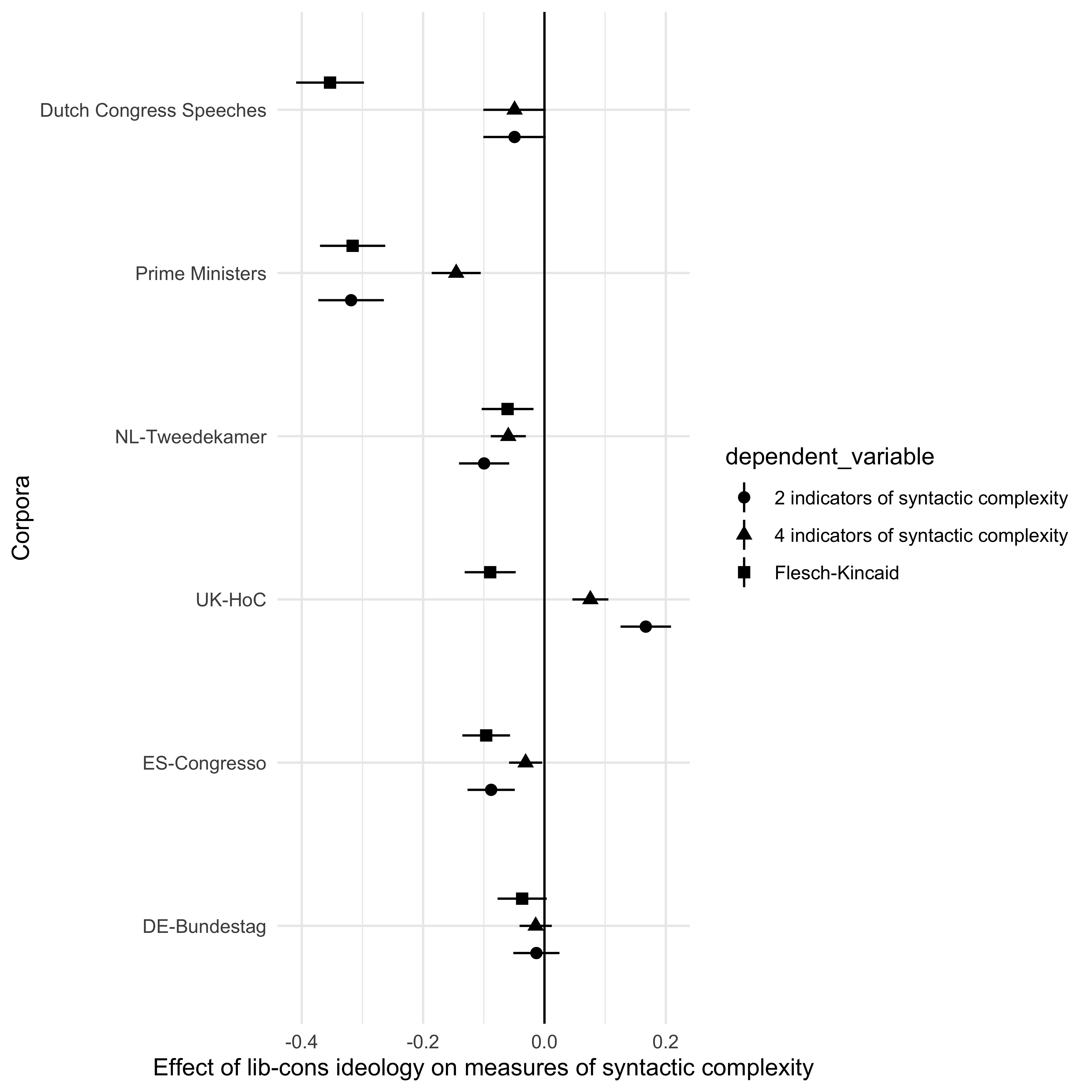

Supplement: S3 File — (PNG) [file pone.0277860.s003.png]

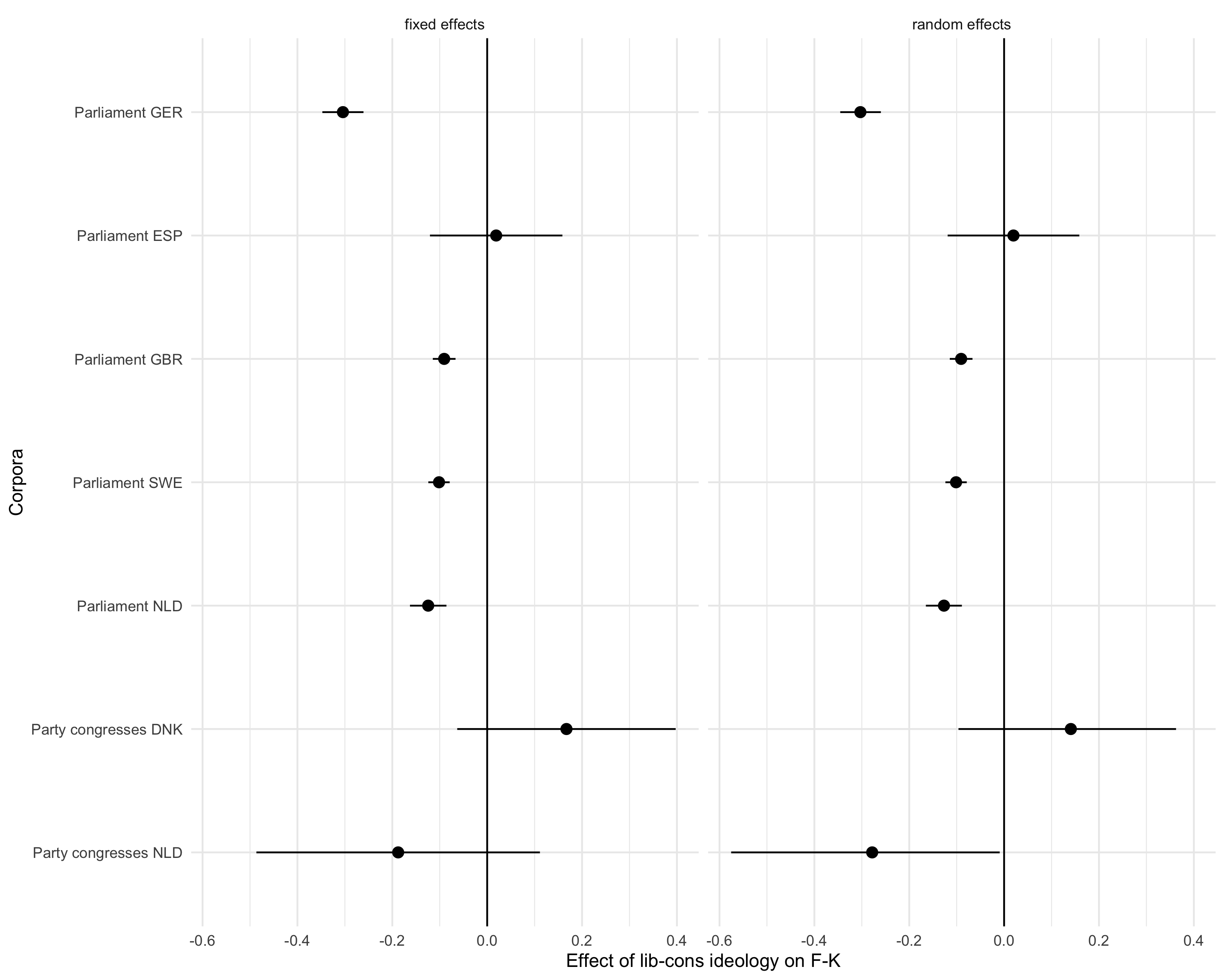

Supplement: S4 File — (PNG) [file pone.0277860.s004.png]

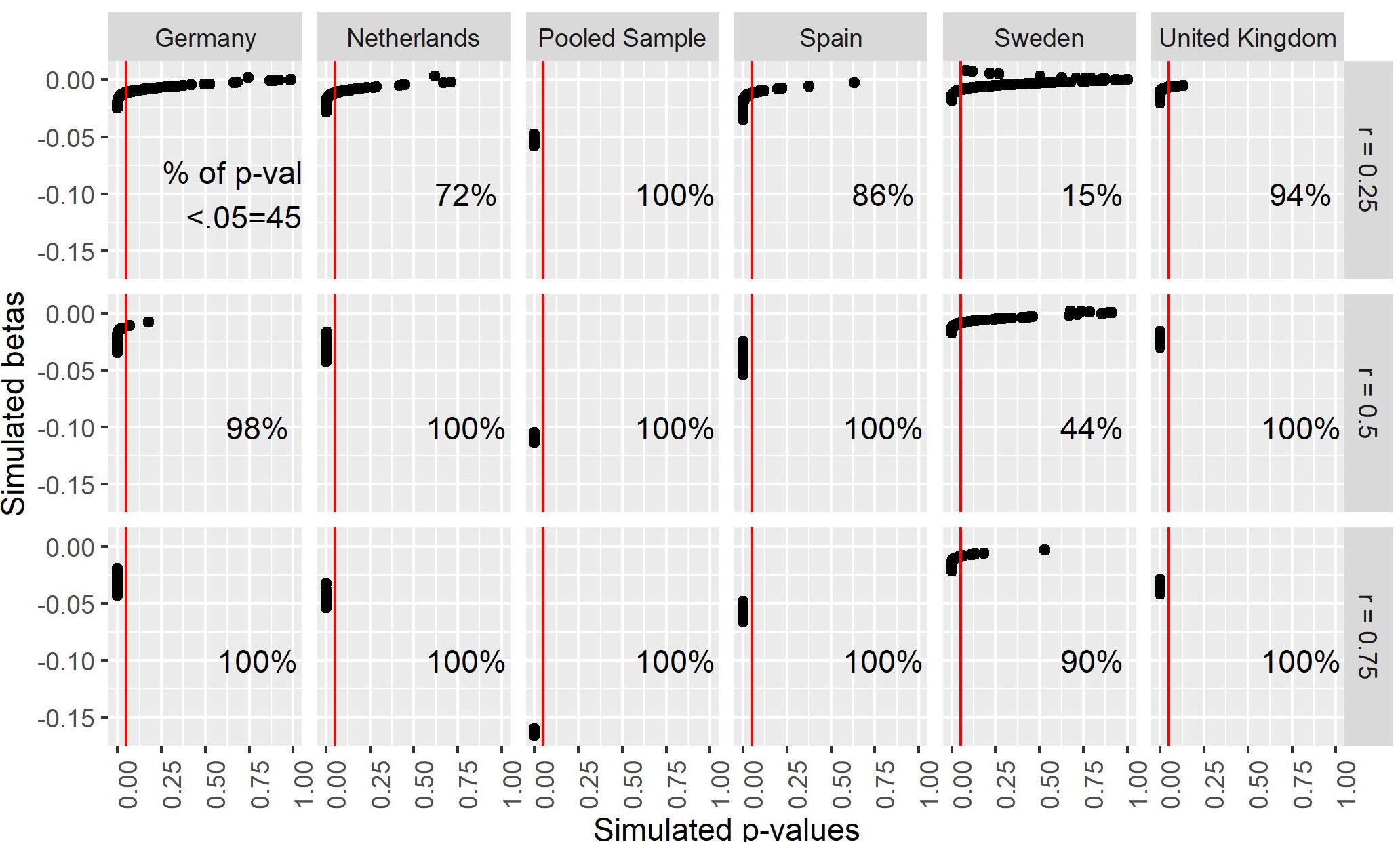

Supplement: S5 File — (JPG) [file pone.0277860.s005.jpg]
